# Incidence Estimates of Acute Q Fever and Spotted Fever Group Rickettsioses, Kilimanjaro, Tanzania, from 2007 to 2008 and from 2012 to 2014

**DOI:** 10.4269/ajtmh.20-1036

**Published:** 2021-12-20

**Authors:** Sruti Pisharody, Matthew P. Rubach, Manuela Carugati, William L. Nicholson, Jamie L. Perniciaro, Holly M. Biggs, Michael J. Maze, Julian T. Hertz, Jo E. B. Halliday, Kathryn J. Allan, Blandina T. Mmbaga, Wilbrod Saganda, Bingileki F. Lwezaula, Rudovick R. Kazwala, Sarah Cleaveland, Venance P. Maro, John A. Crump

**Affiliations:** ^1^ Division of Infectious Diseases and International Health, Department of Medicine, Duke University, Durham, North Carolina;; ^2^ Duke Global Health Institute, Duke University, Durham, North Carolina;; ^3^ Programme in Emerging Infectious Diseases, Duke-National University of Singapore, Singapore;; ^4^ Kilimanjaro Christian Medical Centre, Moshi, Tanzania;; ^5^ Centers for Disease Control and Prevention, Rickettsial Zoonoses Branch, Atlanta, Georgia;; ^6^ Centre for International Health, University of Otago, Dunedin, New Zealand;; ^7^ Department of Medicine, University of Otago, Christchurch, New Zealand;; ^8^ Institute of Biodiversity, Animal Health and Comparative Medicine, College of Medical, Veterinary and Life Sciences, University of Glasgow, Glasgow, United Kingdom;; ^9^ Boyd Orr Centre for Population and Ecosystem Health, Institute of Biodiversity, Animal Health and Comparative Medicine, University of Glasgow, Glasgow, United Kingdom;; ^10^ Kilimanjaro Christian Medical University College, Moshi, Tanzania;; ^11^ Mawenzi Regional Referral Hospital, Moshi, Tanzania;; ^12^ Ministry of Health, Community Development, Gender, Elderly and Children, Dodoma, Tanzania;; ^13^ Sokoine University of Agriculture, Morogoro, Tanzania

## Abstract

Q fever and spotted fever group rickettsioses (SFGR) are common causes of severe febrile illness in northern Tanzania. Incidence estimates are needed to characterize the disease burden. Using hybrid surveillance—coupling case-finding at two referral hospitals and healthcare utilization data—we estimated the incidences of acute Q fever and SFGR in Moshi, Kilimanjaro, Tanzania, from 2007 to 2008 and from 2012 to 2014. Cases were defined as fever and a four-fold or greater increase in antibody titers of acute and convalescent paired sera according to the indirect immunofluorescence assay of *Coxiella burnetii* phase II antigen for acute Q fever and *Rickettsia conorii* (2007–2008) or *Rickettsia africae* (2012–2014) antigens for SFGR. Healthcare utilization data were used to adjust for underascertainment of cases by sentinel surveillance. For 2007 to 2008, among 589 febrile participants, 16 (4.7%) of 344 and 27 (8.8%) of 307 participants with paired serology had Q fever and SFGR, respectively. Adjusted annual incidence estimates of Q fever and SFGR were 80 (uncertainty range, 20–454) and 147 (uncertainty range, 52–645) per 100,000 persons, respectively. For 2012 to 2014, among 1,114 febrile participants, 52 (8.1%) and 57 (8.9%) of 641 participants with paired serology had Q fever and SFGR, respectively. Adjusted annual incidence estimates of Q fever and SFGR were 56 (uncertainty range, 24–163) and 75 (uncertainty range, 34–176) per 100,000 persons, respectively. We found substantial incidences of acute Q fever and SFGR in northern Tanzania during both study periods. To our knowledge, these are the first incidence estimates of either disease in sub-Saharan Africa. Our findings suggest that control measures for these infections warrant consideration.

## INTRODUCTION

Q fever and spotted fever group rickettsioses (SFGR) are common zoonotic causes of febrile illness in sub-Saharan Africa, and both diseases can cause substantial morbidity.^1^^,^^2^ In Tanzania, despite both diseases being common causes of fever, awareness among healthcare providers remains low, and little attention has been focused on measures for disease control or prevention.^3^^,^^4^ Both diseases often have nonspecific presentations, such as fever, myalgia, headache, and fatigue.^5^^,^^6^ An accurate diagnosis is difficult, particularly in resource-limited areas where appropriate diagnostic testing is seldom available.^7^^,^^8^ Under-recognition and under-reporting of cases make it difficult to calculate the reliable disease incidence, which is a key component of disease burden estimates.^9^ To our knowledge, there are no estimates of the incidences of Q fever or SFGR in sub-Saharan Africa, and there are no estimates of global disease burden for either disease.

Our previous studies performed in the Kilimanjaro Region of northern Tanzania demonstrated that Q fever and SFGR are important causes of febrile illness, accounting for 5% and 8% of febrile hospital admissions, respectively.^10^ With the growing awareness of malaria overdiagnosis in tropical low-income and middle-income countries,^11^^,^^12^ epidemiologic characterization of other causes of acute febrile illness is necessary to identify disease prevention priorities and optimize febrile illness treatment algorithms. Although observational studies establishing the frequency of Q fever and SFGR as causes of acute febrile illness might be adequate for improving febrile illness treatment algorithms, the incidence estimates are needed to characterize disease burden and inform the prioritization of these zoonotic infectious diseases in areas where they may be common but are neglected. However, to our knowledge, there have been no incidence estimates of Q fever or SFGR in Tanzania or in sub-Saharan Africa as a whole, and there are no estimates of the global burden of disease for these illnesses.

Although our previous work^10^^,^^13^ has shown that acute Q fever and SFGR are prevalent causes of acute febrile illness in northern Tanzania, this analysis sought to provide age-specific and overall incidence estimates for acute Q fever and SFGR in the Kilimanjaro Region of Tanzania across two periods of febrile illness surveillance. We utilized a widely used hybrid surveillance method that uses facility-based sentinel surveillance to capture cases and adjusts the crude estimate with population-based healthcare utilization survey data.^14^^–^^19^ Providing these incidence estimates is a key step toward understanding the burden of these zoonotic infections in northern Tanzania and deciding how to prioritize disease prevention measures for these and other zoonotic infections.

## MATERIALS AND METHODS

### Study design.

We estimated the incidences of acute Q fever and SFGR by pairing hospital-based sentinel disease surveillance and healthcare utilization surveys in the catchment of the sentinel sites. By measuring healthcare-seeking patterns of those living in the catchment, this hybrid surveillance approach aimed to adjust the observed incidence for cases that did not present to the sentinel surveillance sites. This work was part of a larger project to describe the epidemiology of bacterial zoonoses in northern Tanzania. Incidence estimates using the same hybrid surveillance methods and platform for leptospirosis and brucellosis have already been published.^17^^–^^19^ The prevalence rates of acute Q fever and SFGR among febrile admissions during the first surveillance period of 2007 to 2008 have been published previously.^10^ This present project transforms those findings into incidence estimates and provides the prevalence and incidence estimates for acute Q fever and SFGR during a second surveillance period from 2012 to 2014.^17^^–^^19^

### Hospital-based fever surveillance.

#### Study site.

We conducted sentinel surveillance at Kilimanjaro Christian Medical Center (KCMC) and Mawenzi Regional Referral Hospital (MRRH), which are two hospitals in Moshi, Tanzania.^17^ At the time of these studies, KCMC was a 450-bed hospital and the zonal referral center for multiple regions in northern Tanzania, and MRRH was a 300-bed hospital and the referral center for Kilimanjaro Region. Moshi, the administrative capital of the Kilimanjaro region of Tanzania, consists of the Moshi Municipal District and Moshi Rural District. Moshi is situated at an elevation of approximately 890 m and has a tropical climate, with a short rainy season from October through December and a long rainy season from March through May.^20^ Aside from the urban areas of Moshi Municipal District, the region is rural and its inhabitants are primarily involved in cultivation and small-holder farming.^17^

#### Study population.

During the first study period of 2007 to 2008, only febrile inpatients were enrolled. Participants younger than 13 years were eligible if they had a history of fever within the previous 48 hours, an axillary temperature 37.5°C or higher, or rectal temperature 38.0°C or higher. Participants 13 years or older were eligible if they had an oral temperature 38.0°C or higher. Screening occurred within 24 hours of admission to hospital. During the second study period of 2012 to 2014, both febrile inpatients and febrile outpatients were enrolled. Inpatients were screened within 24 hours of admission and eligible if they had a history of fever within the previous 72 hours or if they had a tympanic temperature 38.0°C or higher at screening. Outpatients were eligible if they had a tympanic temperature 38.0°C or higher at the time of screening.^17^^–^^19^

#### Study procedures.

Study procedures have been described in detail previously^10^^,^^17^^–^^19^ but are briefly reviewed here. Prospective enrollment of febrile patients occurred Monday through Friday at both study sites. From September 17, 2007 through August 31, 2008, consecutive febrile pediatric and adult inpatients at KCMC and consecutive febrile adult inpatients at MRRH were offered enrollment. From February 20, 2012 through May 28, 2014, consecutive febrile adult inpatients at KCMC and consecutive febrile adult and pediatric inpatients at MRRH were offered enrollment. Additionally, during the second study period, the study team offered enrollment to every second febrile adult or pediatric patient presenting to the outpatient department at MRRH. Demographic data, including the participant’s district and village of residence, were collected. Acute serum was collected at enrollment, and participants were asked to return 4 weeks later for the collection of convalescent serum. Travel reimbursement was provided to participants who attended the follow-up visit. For participants who did not attend the scheduled follow-up, study staff attempted to contact them first by phone and then, if necessary, by a field worker home visit to encourage attendance before closure of the follow-up window at 6 weeks after enrollment. Participants were included in the case count for acute Q fever and SFGR if they had paired (i.e., acute and convalescent) sera available for serologic testing. Unavailability stemmed from either the sample not being collected (e.g., participant did not attend the follow-up visit) or insufficient sample collection either at enrollment or during follow-up.

#### Laboratory diagnosis and case definitions.

Acute and convalescent serum samples were sent to the United States Centers for Disease Control and Prevention Rickettsial Zoonoses Branch for serologic analysis for Q fever and SFGR by indirect immunofluorescence assay (IFA).^13^^,^^21^ For the 2007 to 2008 study period, samples were tested from 2009 to 2010; for the 2012 to 2014 study period, samples were tested from 2016 to 2019. For the SFGR IFA, the antigens used were *Rickettsia conorii* for the 2007 to 2008 study period and *Rickettsia africae* for the 2012 to 2014 study period. To minimize variations, all serology was performed by the same respective laboratorian for each study period, and the same respective lots of IFA antigen for each pathogen were used for all testing during each respective study period. Variations in stocks of IgG antibody conjugates and positive controls and negative controls for the IFA were kept to a minimum. Any new reagent lot for controls underwent parallel testing for quality assurance and to ensure the control endpoint titers were consistent. Acute and convalescent samples were tested in tandem to minimize any potential misclassification because of batch effect assay variability.

Confirmed acute cases of Q fever were defined a four-fold or greater increase between acute and convalescent antibody titers for *C. burnetii* phase II antigen.^22^ Confirmed acute cases of SFGR were defined as a four-fold or greater increase between acute and convalescent antibody titers for *Rickettsia conorii* (2007–2008) or *Rickettsia africae* (2012–2014) antigens.^23^

### Healthcare utilization survey.

A healthcare utilization survey was performed in the Moshi Municipal (population 184,292 in 2012) and Moshi Rural (population 466,737 in 2012)^24^ Districts of the Kilimanjaro Region of Tanzania from 13 June 2011 through 22 July 2011, as previously described.^17^^,^^25^ The sampling frame and sample size calculations for the healthcare utilization surveys were adapted from the World Health Organization Expanded Program of Immunization methods, which ultimately generated a sampling frame of 30 cluster units in this case administrative wards and 27 households per cluster.

Survey respondents were adults who identified as the primary caregiver of each selected household. Hypothetical febrile illness scenarios were presented to respondents, and questions were asked to identify their healthcare-seeking behaviors to ascertain the proportion of respondents likely to present to KCMC or MRRH in the event of a febrile illness. Questions such as “What will you do if you have a fever?” and “What will you do if you have a fever for 3 days or more?” were asked. We selected the latter as our primary question to reflect the healthcare-seeking behavior of respondents because it presented a febrile illness scenario that was both broad in scope and specific enough to suggest an illness that was not self-limited or mild.

### Incidence calculation.

**Figure 1.**
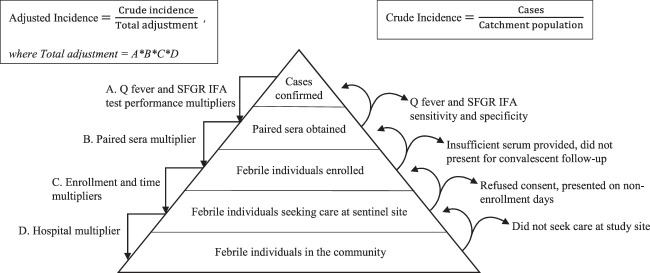
Sentinel surveillance pyramid showing incomplete capture of Q fever and spotted fever group rickettsioses (SFGR) cases in a surveillance catchment. The adjustment multipliers used to correct for case underascertainment are shown (left side) along with explanations for each (right side). Indirect immunofluorescence assay (IFA) performance multipliers are derived from the literature. Paired sera, enrollment, and time multipliers are derived from study documentation. The hospital multiplier is derived from responses to the healthcare utilization survey. This figure has been expanded and modified from those of Crump et al.^63^ and Andrews et al.^16^

Incidence was estimated based on the absolute number of participants who met the case definition for acute Q fever or SFGR. Then, multipliers were created to adjust for factors that would result in our surveillance platform underascertaining cases in the catchment area (Figure 1). All multipliers were the multiplicative inverse of the relevant proportions (Table 1). For example, because enrollment occurred during only 5 of the 7 days of the week, the time multiplier was 7/5 or 1.4. Because not all enrolled participants were able to provide acute and convalescent serum samples, a paired sera multiplier for each disease was calculated by dividing the total number of enrolled participants by the number of participants from who we were able to collect and test paired sera. The hybrid surveillance multiplier method has been previously described in further detail.^15^^,^^16^ Consistent with the incidence classification used for other infectious diseases,^26^^,^^27^ incidences were classified as low, moderate, high, and very high and corresponded to incidence ranges of less than 10 cases per 100,000 persons annually, 10 to less than 100 cases per 100,000 persons annually, 100 to less than 500 cases per 100,000 persons annually, and 500 or more cases per 100,000 persons annually, respectively.

**Table 1:** Derivation of multipliers to estimate incidences of Q fever and spotted fever group rickettsioses (SFGR) in the Moshi Rural and Moshi Urban Districts, Kilimanjaro Region, Tanzania

	Multiplier equation	Study period					
		2007–2008				2012–2014	
Q fever IFA sensitivity multiplier	1/sensitivity	1/1		1		1/1	1
Q fever IFA specificity multiplier	Specificity	0.95		0.95		0.95	0.95
SFGR IFA sensitivity multiplier	1/sensitivity	1/0.94		1.06		1/0.94	1.06
SFGR IFA specificity multiplier	Specificity	1		1		1	1
KCMC multiplier	No. of households interviewed/no. households seeking care at KCMC for fever ≥ 3 days						
Age 0–4 years		198/17		11.65		198/17	11.63
Age 5–14 years		361/10		361/10		361/10	36.10
Age ≥ 15 years		810/35		23.14		810/35	23.14
MRRH multiplier	No. of households interviewed/no. households seeking care at MRRH for fever ≥ 3 days						
Age 0–4 years		198/67		2.96		198/67	2.96
Age 5–14 years		361/137		2.64		361/137	2.64
Age ≥ 15 years		810/299		2.71		810/299	2.71
Paired sera multiplier	No. of patients included in the study/no. of patients included in the study with paired sera	Q fever	SFGR	Q fever	SFGR		
Age 0–4 years		256/143	256/124	1.79	2.06	430/177	2.43
Age 5–14 years		59/45	59/42	1.31	1.40	111/87	1.28
Age ≥ 15 years		274/156	274/141	1.76	1.94	573/377	1.52
Time multiplier	No. of days in a week/no. of enrollment days per week	7/5		1.40		7/5	1.40
Enrollment multiplier	No. of eligible patients/no. of patients enrolled in fever surveillance	1,310/870		1.51		2,962/1,414	2.09
Study duration adjustment	No. days per year/study duration (in days)	365/349		1.05		365/828	0.44

IFA = indirect immunofluorescence assay; KCMC = Kilimanjaro Christian Medical Centre; MRRH = Mawenzi Regional Referral Hospital; SFGR = spotted fever group rickettsioses.

#### Derivation of multipliers.

The following multipliers were calculated and used for incidence estimation: hospital multipliers for KCMC and MMRH to reflect the healthcare-seeking behavior of Moshi residents and to account for cases potentially missed because of presentation at facilities not undergoing surveillance, derived from the proportion of respondents who selected either hospital as their first or second choice in response to the question “In light of the hypothetical febrile illness scenario, what will you do if you have fever for 3 days or more?”; an enrollment multiplier to account for patients who were eligible but not enrolled because the residence of those who declined to participate was not recorded (the enrollment multiplier calculations included participants residing in both study and nonstudy districts); a paired sera multiplier to account for participants who did not provide convalescent samples 4 to 6 weeks after acute samples were collected; study duration adjustment to calculate the annual incidence from studies that were not precisely 1 year in duration; a time multiplier to account for enrollment during only 5 days of the week; and sensitivity and specificity multipliers to account for the sensitivity and specificity of the IFA for acute Q fever (100% and 95%, respectively)^28^^,^^29^ and SFGR (94% and 100%, respectively)^30^^,^^31^ reported in the literature.

#### Population denominators.

Incidences were calculated by age group as follows: 0 to 4 years, 5 to 14 years, and 15 years or older. For the first study period, we averaged age-specific proportions and overall population totals from the 2002 and 2012 Population and Housing Censuses produced by the National Bureau of Statistics of the United Republic of Tanzania to estimate age-specific population totals for 2007 to 2008 incidence estimates.^24^^,^^32^ For 2012 to 2014 incidence estimates, we used age-specific population totals provided by the 2012 census.

#### Uncertainty range calculation.

To describe the uncertainty regarding the incidence point estimates, we modeled plausible upper and lower limits of incidence by using the upper and lower limits of the core parameters of our estimate model: disease prevalence informed by variable diagnostic performance and cases identified in the enrolled populations and healthcare-seeking behavior. We used the binomial exact 95% confidence intervals (CI) for the observed prevalence for both Q fever and SFGR, the binomial exact 95% CI for the observed proportion of healthcare utilization survey respondents who listed KCMC or MRRH as their first or second choice, and the plausible sensitivity and specificity ranges of paired sera for IFA for both diseases based on the published literature. For acute Q fever, we used a specificity range of 95.3%^28^ to 96.0%^33^ and a sensitivity range of 86.1%^34^ to 100%.^28^^,^^29^^,^^34^ For SFGR, we used a specificity of 100%^31^ and a sensitivity range of 83.0% to 94.0%.^30^^,^^35^ These alternative values were used to calculate the upper and lower limits of incidence for both diseases, producing an uncertainty range. When comparing two incidence estimates, an observation of a true difference was defined as estimates with uncertainty ranges that did not overlap.

#### Sensitivity analysis of incidence estimates.

We anticipated that responses to the healthcare utilization survey might vary depending on the specific febrile illness scenario presented to the respondents; therefore, we examined the variability in incidence estimates introduced by using hospital multipliers based on responses to different scenarios. We performed a one-way sensitivity analysis by varying hospital multipliers according to answers to an alternative question, “What will you do if you have a fever?,” on the healthcare utilization survey describing a scenario of fever with unspecified duration.

### Statistical analysis.

Data were entered using the OpenText TeleForm system (Waterloo, Canada) into an Access database (Microsoft Corporation, Redmond, WA). Incidence calculations were performed using Microsoft Excel 2016 (Microsoft Corporation) spreadsheets.

### Research ethics.

All study participants provided written informed consent. For participants younger than 18 years, a parent or guardian provided written informed consent. Additionally, for those 12 years to younger than 18 years, written assent was required. All data were derived from the 2007 to 2008 study protocols and the 2012 to 2014 study protocols, both of which were approved by the Kilimanjaro Christian Medical University College Research Ethics Committee, the Tanzania National Institute for Medical Research National Ethics Coordinating Committee, and the Institutional Review Board of Duke University Hospital System.

## RESULTS

### Hospital-based fever surveillance.

During 2007 to 2008, of the 1,310 eligible patients, 870 (66.4%) were enrolled. Of those participants, 589 (67.7%) were from the catchment area of Moshi Municipal and Moshi Rural Districts. Among participants from the catchment area, 256 (43.5%) were younger than 5 years, 59 (10.0%) were 5 to 14 years old, and 274 (46.5%) were 15 years or older. The median age was 8 years (range, < 1–96) years, and 298 (50.6%) participants were female. Among the 589 participants residing in the catchment area, 344 (58.4%) and 307 (52.1%) had paired sera tested for Q fever and SFGR, respectively. Of those with tested paired sera, 16 (4.7%) and 27 (8.8%) fulfilled the case definitions for acute Q fever and SFGR, respectively. The median age of patients with Q fever was 25 years (range, < 1–72 years), and 11 (68.8%) Q fever patients were female. The median age of SFGR patients was 23 years (range, < 1–76 years), and 11 (40.7%) SFGR patients were female.

During 2012 to 2014, of the 2,962 patients who were eligible for enrollment, 1,414 (47.7%) participated in the study. Of those participants, 1,114 (78.8%) were from the catchment area of Moshi Municipal and Moshi Rural Districts. Among participants from the catchment area, 430 (38.6%) were younger than 5 years old, 111 (10.0%) were 5 to 14 years old, and 573 (51.4%) were 15 years or older. The median age was 18 years (range, < 1–100 years); 592 (53.1%) were female, and 473 (42.5%) were outpatients. Among the 1,114 participants residing in the catchment area, 641 (57.5%) had paired sera tested for both Q fever and SFGR; of these, 258 (40.2%) were outpatients. Of those with tested paired sera, 52 (8.1%) and 57 (8.9%) fulfilled the case definitions for acute Q fever and SFGR, respectively. Of these, 17 (32.7%) and 23 (40.4%) of the confirmed acute Q fever and SFGR cases were observed in outpatients, respectively. The median age of Q fever patients was 12.5 years (range, < 1–61 years), and 26 (50.0%) Q fever patients were female. The median age of SFGR patients was 5 years (range, < 1–70 years), and 32 (56.1%) SFGR patients were female.

### Healthcare utilization survey.

The 810 households sampled included 3,919 household members. All households had at least one member 15 years or older, 361 (44.6%) had at least one member 5 to 14 years of age, and 198 (24.4%) had at least one member 0 to 4 years of age. Table 1 includes responses to the question, “What will you do if you have a fever for 3 days or more?” and the calculations for hospital multipliers.

#### Incidence calculation and uncertainty range.

**Figure 2.**
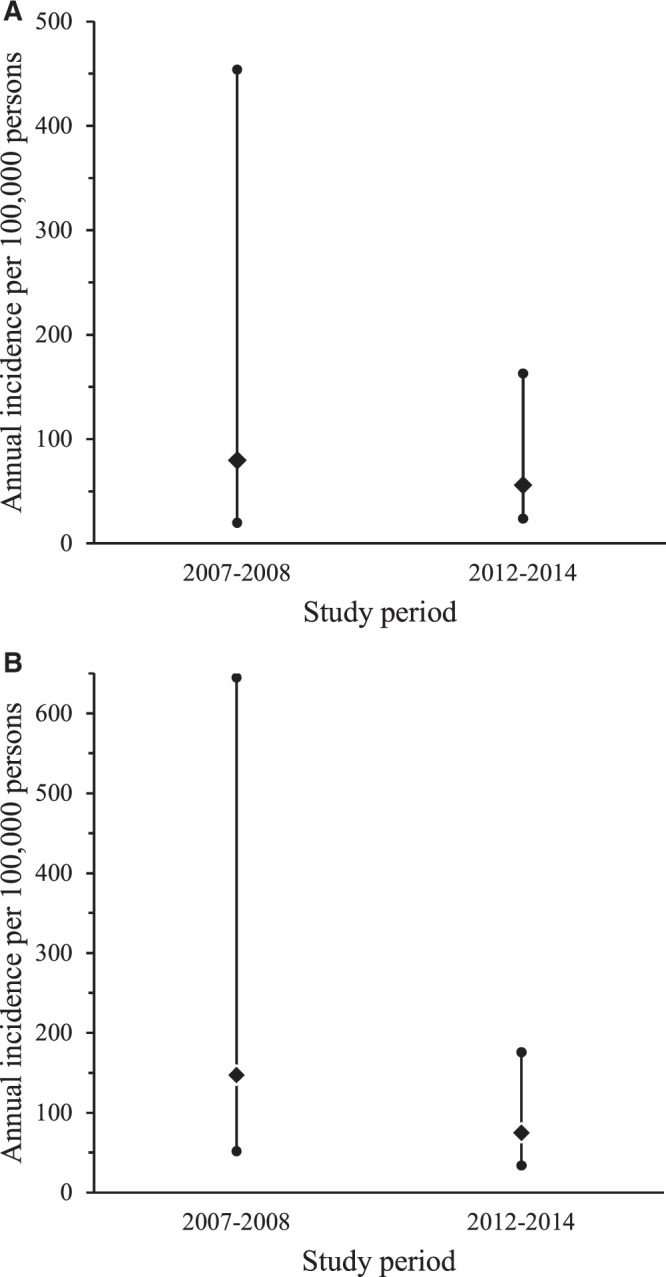
Incidence point estimates and uncertainty ranges for Q fever (**A**) and spotted fever group rickettsioses (SFGR) (**B**) in Kilimanjaro, Tanzania, from 2007 to 2008 and from 2012 to 2014.

Adjustment multipliers were calculated as described in Table 1. The application of multipliers to confirmed cases to estimate the overall incidences of acute Q fever and SFGR is shown in Supplemental Tables 1 and 2. For the study period from 2007 to 2008, we estimated the overall annual incidences of acute Q fever and SFGR to be 80 (uncertainty range, 20–454) cases and 147 (uncertainty range, 52–645) cases per 100,000 persons, respectively. For the study period from 2012 to 2014, we estimated the overall annual incidences of acute Q fever and SFGR to be 56 (uncertainty range, 24–163) cases and 75 (uncertainty range, 34–176) cases per 100,000 persons, respectively. Figure 2 shows the incidence point estimates and uncertainty ranges.

As shown in Table 2, during the study period from 2007 to 2008, the incidence of SFGR for children 0 to 4 years old was higher than the incidence for those 15 years or older, without overlapping uncertainty ranges. During the study period from 2012 to 2014, the incidence of acute Q fever in children 0 to 4 years old was higher than that for children 5 to 14 years old, and the incidence of SFGR for children 0 to 4 years old was higher than that for the other two age groups and the overall population, also without overlapping uncertainty ranges (Table 2).

**Table 2:** Incidence estimates per 100,000 persons annually by age category for acute Q fever and spotted fever group rickettsioses (SFGR), Moshi Municipal and Moshi Rural Districts, Tanzania, 2012–2014

	2007–2008 Incidence (uncertainty range)	2012–2014 Incidence (uncertainty range)
Q fever		
0–4 years	241 (61–1,125)	250 (115–641)
5–14 years	51 (5–578)	19 (7–61)
≥15 years	62 (19–233)	37 (14–120)
Overall	80 (20–454)	56 (24–163)
SFGR		
0–4 years	931 (346–2,854)	390 (195–780)
5–14 years	61 (6–669)	6 (1–36)
≥15 years	26 (14–145)	48 (19–126)
Overall	147 (52–645)	75 (34–176)

#### Sensitivity analysis of incidence estimates.

A one-way sensitivity analysis using an alternative hypothetical scenario, “What will you do if you have a fever?,” to adjust for case underascertainment produced the following incidence estimates: during the 2007 to 2008 and 2012 to 2014 study periods, acute Q fever annual incidences were estimated to be 209 cases and 152 cases per 100,000 persons, respectively, and during the 2007 to 2008 and 2012 to 2014 study periods, SFGR annual incidences were estimated to be 372 cases and 156 cases per 100,000 persons, respectively.

## DISCUSSION

We found moderate incidence point estimates for acute Q fever in Moshi Municipal and Moshi Rural Districts in the Kilimanjaro Region of Tanzania during both the 2007 to 2008 and 2012 to 2014 study periods. We found a high incidence point estimate of SFGR during the 2007 to 2008 study period, and a moderate incidence point estimate of SFGR during the 2012 to 2014 study period in the same region. During both study periods, we observed high incidences of both acute Q fever and SFGR among those 0 to 4 years of aging, which is a finding that merits further study. To our knowledge, our estimates are the first incidence estimates of Q fever and of SFGR in sub-Saharan Africa to be reported, and they begin to characterize the burden of these illnesses in the region. In the context of prior prevalence studies that have described Q fever and SFGR as important causes of febrile illness in sub-Saharan Africa,^1^^,^^2^ our findings indicate that Q fever and SFGR are relevant zoonotic public health concerns that merit further attention and investigation for disease control and prevention.

Our observation of the moderate and stable incidence of acute Q fever in northern Tanzania is likely relevant to other settings in sub-Saharan Africa, where *C. burnetii* infections have been described as common. A number of studies characterize *C. burnetii* exposure through seroprevalence in community-based surveys or Q fever disease prevalence in febrile study populations in sub-Saharan Africa.^36^ In northeastern Kenya, acute Q fever was diagnosed in 173 (16.2%) of 1,067 febrile patients.^37^ In Dar es Salaam, Tanzania, 7 (4.7%) of 150 pregnant women had serologic evidence of exposure to *C. burnetii*.^38^ In Cameroon, 6 (9.2%) of 65 patients were diagnosed with *C. burnetii* pneumonia.^39^ High seroprevalences of *C. burnetii* described in southern, east, and west Africa^4^ indicate that it is a common infection in these regions, thereby suggesting, in the absence of etiological data, that *C. burnetii* is likely a cause of acute febrile illness in these regions.

Similarly, our observation of the moderate to high endemic incidence of SFGR in northern Tanzania may suggest substantial SFGR incidences in other parts of sub-Saharan Africa. Multiple etiology studies performed in east and west Africa demonstrate that SFGR is a prevalent cause of febrile illness, with study prevalence rates ranging from 0.07% to 8% of febrile participants.^13^^,^^40^^,^^41^ Furthermore, SFGR is the second commonest cause of febrile illness among travelers returning from sub-Saharan Africa.^42^^,^^43^ The prevalence of SFGR among febrile patients and seroprevalence in community surveys suggest potentially high incidences in multiple countries across sub-Saharan Africa.^2^^,^^38^^,^^40^^,^^44^ Although *R. africae*, *R. conorii*, and *Rickettsia felis* have been identified as causes of fever in sub-Saharan Africa,^45^^–^^47^ it is unclear which species of SFGR are predominant in east Africa. Consequently, although several species of SFGR can cause severe and fatal disease,^48^^,^^49^ including *R. conorii*,^50^ it remains unclear whether SFGR could be causing fatal febrile illness in east African countries. Few individual instances of death from SFGR in sub-Saharan Africa have been reported previously.^51^ Our study methods did not allow identification of fatal cases, and, to our knowledge, case fatality attributable to SFGR has not been described in any observational study of febrile cohorts. Nonetheless, the moderate to high incidence we estimated for SFGR heightens the need to further characterize the predominant *Rickettsia* species in east Africa.

We observed high incidences of acute Q fever in children 0 to 4 years of age during both study periods, and we observed very high and high incidences of SFGR from 2007 to 2008 and from 2012 to 2014, respectively (Table 2). Uncertainty ranges of each of these incidence estimates did not overlap with the uncertainty ranges of at least one other age group, suggesting that incidences among young children may be truly higher than those of other age groups or the overall population. Other studies have demonstrated these illnesses are common in children: in western Kenya, 25 (8.9%) and 63 (22.4%) of 364 febrile children 1 to 12 years old were found to have acute Q fever and SFGR, respectively.^1^ Studies have also reported higher SFGR prevalence and incidence among children younger than 10 years old in Mexico and the United States, respectively.^48^^,^^52^ Fatal pediatric cases of SFGR have been reported in multiple countries, including the United States, Brazil, and Colombia.^49^^,^^52^^,^^53^ Variations in incidence by age may reflect differences in susceptibility to disease, in exposure, or in actual health-seeking behavior.

For Q fever, however, the current body of literature suggests that young children are less susceptible to disease, and the illness is thought to present more mildly in children and adolescents than in adults.^54^^–^^56^ During a 1983 outbreak in Switzerland, children younger than 15 years accounted for 80 (19%) of 415 seropositive cases, but only 10 (5%) of 191 symptomatic cases and none of 8 hospitalized cases.^57^ A study in Greece showed that younger children were significantly less likely to become symptomatic after infection than adolescents aged 11 to 14 years, and the average fever duration among the pediatric cohort was shorter than the fever duration reported for adults with Q fever.^58^ This literature does not necessarily contradict our findings of the higher incidence for young children. Our broad inclusion criteria may have reduced selection bias toward severe disease and led to enrollment of milder pediatric Q fever cases. Disproportionately increased exposure to *C. burnetii* in young children in sub-Saharan Africa might also lead to a higher incidence in this age group. Community surveys in Niger, Ghana, and Gambia showed a disproportionately higher seroprevalence of *C. burnetii* among young children.^59^^–^^61^

For SFGR, increased exposure and increased susceptibility to disease could explain the higher incidence for young children. In northern Mexico, children were found to have a disproportionately higher prevalence of antibodies *Rickettsia* species compared with adults, and this was attributed to greater exposure to dogs carrying infected ticks.^48^ The same study found a case fatality ratio of up to 30% among children younger than 10 years diagnosed with SFGR compared with the aggregate case fatality ratio of 8% reported for the general population of the state.^48^ This suggests that children might be more susceptible to severe SFGR infections. Studies performed in sub-Saharan Africa have also found a high prevalence of antibodies to *Rickettsia* species among children. The community-based study in Niger found serologic evidence of prior exposure to *R. conorii* in 31 (17.5%) of 177 children younger than 5 years.^59^ Although it is plausible that children in our predominantly rural catchment area experience greater exposure to *C. burnetii* and *Rickettsia* species, the modes of transmission to children in our setting remain poorly understood. Given the agrarian ecology of the catchment area, with livestock present in both rural and urban environments, participants in our study may have experienced more direct and frequent contact with livestock infected with *Coxiella burnetii* or dogs infested with *Rickettsia*-infected ticks. It is plausible that children 0 to 4 years of age may have substantial exposure to such household livestock and animals because they spend more time at home. Whether children would be more susceptible to wind-borne transmission of *C. burnetii* is less clear. Alternative explanations for the high incidence we observed for children 0 to 4 years of age would include differences in health-seeking behavior between age groups; in other words, parents may seek care more frequently for younger children with milder illness, which could bias our study toward more case detections in younger children. Our healthcare utilization survey found that few respondents selected KCMC or MRRH as their choice of healthcare facility for febrile children 5 to 14 years old, perhaps reflecting that older children may be less likely to be brought to a large hospital for febrile illnesses. Overall, although higher incidences of acute Q fever and SFGR in young children are plausible, our results should be interpreted with caution, and further studies are needed to confirm whether young children in the Kilimanjaro Region comprise a high-risk population for these diseases.

We attempted to measure uncertainty regarding our incidence point estimates by accounting for the uncertainty of three key parameters of the hybrid surveillance method: the precision of the observed proportion of participants who were found to have cases of acute Q fever or SFGR; the plausible range of sensitivity and specificity for the diagnostic tests; and the precision of the observed proportion of respondents reporting that they would seek healthcare at the study sentinel facilities in the event of fever. The former two factors introduced minimal uncertainty, but the latter factor, represented by hospital multipliers, introduced much more uncertainty, resulting in higher incidence point estimates and expansion of the corresponding uncertainty ranges. Our sensitivity analysis revealed that incidence estimates from a hybrid surveillance system such as ours can vary substantially depending on how healthcare-seeking behavior is assessed. Use of hospital multipliers derived from the healthcare utilization survey question, “What will you do if you have a fever?” resulted in higher incidences and much wider uncertainty ranges than the question “What will you do if you have a fever for 3 days or more?” did. However, the sensitivity analysis was most valuable for demonstrating that investigators quantifying the incidence with hybrid surveillance should carefully select their primary question and design several questions to accurately characterize healthcare-seeking behavior within the selected population.

The multiplier method used during our hybrid surveillance was predicated based on several key assumptions: the same proportion of cases existed among those who did not have paired sera collected compared with those who had sufficient paired sera for testing; eligible but nonenrolled patients had the same prevalence of acute Q fever and SFGR as those who were enrolled; those presenting to surveillance sites were representative of the respondents to our healthcare utilization study; survey questions accurately captured healthcare-seeking behavior in our catchment; and the hypothetical behavior represents the actual behavior. Additional limitations of our study should be noted, particularly differences in eligibility criteria and recruitment sites between both study periods. We did not evaluate other aspects of disease burden, such as attributed disability and mortality. The former was beyond the scope of our study, requiring more intensive follow-up and data collection. The latter was not possible because we required paired sera IFA to identify cases, which inherently requires that the participants be alive 4 to 6 weeks after the febrile illness. Although the diagnostic tests used for Q fever were consistent across both study periods, the SFGR IFA used *R. conorii* antigen during the first study period and *R. africae* antigen during the second study period. One could speculate that because *R. africae* is thought be the predominate rickettsial species for SFGR in sub-Saharan Africa,^45^ the use of *R. africae* antigen during the second study period could have been a more sensitive diagnostic approach (i.e., more SFGR cases detected than if *R. conorii* antigen had been used). Nevertheless, given the strong serologic cross-reactivity between *R. conorii* and *R. africae* antigens,^62^ it is unlikely that the use of *R. conorii* versus *R. africae* had a large impact on the SFGR case detection during either study. Our incidence estimate approach included adjustment to account for diagnostic sensitivity and diagnostic specificity of the IFA assays. For SFGR, we relied on sensitivity and specificity estimates for *R. rickettsii* because published reports of sensitivity and specificity for either *R. africae* or *R. conorii* were not found. Utilizing *R. rickettsii* IFA performance was justified because of the strong cross-reactivity among the *Rickettsia* species that comprise SFGR. Overall, the adjustments made to account for diagnostic performance, a multiplier of 1.06 to account for the reported 94% sensitivity of IFA for *R. rickettsii*^30^ and a multiplier of 1.00 (i.e., no adjustment of incidence estimates) to account for the reported 100% specificity of IFA for *R. rickettsii*,^31^ were small.

Several robust aspects of our hybrid surveillance should be highlighted. Our results are based on two study periods separated by 4 years with more than 3 years of surveillance. Our diagnostic testing and case definitions have high sensitivities and specificities for both diseases.^28^^–^^31^ Although the gold standard for estimating incidence is active case-finding at the population level, it is often cost-prohibitive and resource-prohibitive in many settings, including northern Tanzania. However, crude incidence estimates derived from sentinel surveillance alone can underestimate the true incidence. Therefore, hybrid surveillance using prospective sentinel surveillance along with the use of healthcare utilization surveys are more practical than active surveillance case-finding and more accurate than crude incidence estimates without adjustment for healthcare-seeking behavior.^16^^,^^26^ Additionally, we incorporated uncertainty ranges for our incidence point estimates to provide a transparent and rigorous approach to estimating incidence.

In conclusion, we found a moderate to high incidence of acute Q fever and a moderate to high incidence of SFGR during the periods 2007 to 2008 and 2012 to 2014 in Moshi Municipal District and Moshi Rural District of the Kilimanjaro Region of northern Tanzania. The incidences of acute Q fever and SFGR are likely substantial in other sub-Saharan African countries, especially where studies have already described these diseases as prevalent causes of febrile illness. More research is needed to describe the burden of these diseases in terms of not only incidence but also disability and mortality. Although further studies are required to characterize the sources, reservoirs, modes of transmission, and risk factors for human infections, our observation of a substantial endemic incidence in northern Tanzania suggests that Q fever and SFGR should be in the national as well as international policy agendas for zoonotic disease control.

## Supplemental Material


Supplemental materials
