# Supplementary material for: Incidence Estimates of Acute Q Fever and Spotted Fever Group Rickettsioses, Kilimanjaro, Tanzania, from 2007 to 2008 and from 2012 to 2014
Source: Am J Trop Med Hyg. 2021 Dec 20;106(2):494–503. doi: 10.4269/ajtmh.20-1036 (PMC8832940; doi:10.4269/ajtmh.20-1036)
Supplement: Supplementary file 1 [file tpmd201036.SD1.pdf]

Supplemental Table 1. Calculation of incidence of acute Q fever and spotted fever group rickettsioses (SFGR), Moshi Municipal and Moshi Rural Districts, Tanzania 2007-2008.

Age group, years	KCMC crude cases	Q fever IFA performance multiplier*	KCMC hospital multiplier	KCMC adjusted cases	MRRH crude cases	Q fever IFA performance multiplier*	MRRH hospital multiplier	MRRH adjusted cases	Total adjusted cases‡	Paired sera multiplier	Enrollment logistics multiplier§	Estimated annual cases	Population	Annual incidence per 100,000 persons
0-4	4	0.95	11.65	44	N/A	0.95	2.96	N/A	44	1.79	2.20	175	72663	241
5-14	1	0.95	36.10	34	N/A	0.95	2.64	N/A	34	1.31	2.20	99	195442	51
≥15	4	0.95	23.14	88	7	0.95	2.71	18	53	1.76	2.20	206	329994	62
Overall												480	598099	80
Age group, years	KCMC crude cases	SFGR IFA performance multiplier†	KCMC hospital multiplier	KCMC adjusted cases	MRRH crude cases	SFGR IFA performance multiplier†	MRRH hospital multiplier	MRRH adjusted cases	Total adjusted cases‡	Paired sera multiplier	Enrollment logistics multiplier§	Estimated annual cases	Population	Annual incidence per 100,000 persons
0-4	12	1.06	11.65	149	N/A	1.06	2.96	N/A	149	2.07	2.20	677	72663	931
5-14	1	1.06	36.10	38	N/A	1.06	2.64	N/A	38	1.40	2.20	119	195442	61
≥15	0	1.06	23.14	0	14	1.06	2.71	40	20	1.94	2.20	86	329994	26
Overall												882	598099	147

* Q fever IFA performance multiplier was the specificity (95%) multiplied by the inverse of the sensitivity (100%).

† SFGR IFA performance multiplier was the specificity (100%) multiplied by the inverse of the sensitivity (94%).

‡ Total adjusted cases for those ≥15 years old was the sum of KCMC and MRRH cases divided by 2 to correct for recruitment of participants from two sentinel sites. Participants who were 0-4 years old and 5-14 years old were only recruited from one sentinel site, KCMC. Thus, total adjusted cases for the two age categories was the same as KCMC adjusted cases for those categories.

§ Enrollment logistics multiplier was the product of three individual multipliers related to enrollment procedures: time multiplier, enrollment multiplier, and study duration multiplier. For 2007-2008, time multiplier was 1.4, enrollment multiplier was 1.51, and study duration adjustment was 1.05. The enrollment logistics multiplier was 2.20 and not 2.21 because it was the product of each individual multiplier prior to rounding.

Abbreviations: IFA, indirect immunofluorescence assay; SFGR, spotted fever group rickettsioses; KCMC, Kilimanjaro Christian Medical Centre; MRRH, Mawenzi Regional Referral Hospital

Supplemental Table 2. Calculation of incidence of acute Q fever and spotted fever group rickettsioses (SFGR), Moshi Municipal and Moshi Rural Districts, Tanzania 2012-2014. Based on the question, 'What will you do if you have fever for ≥ 3 days?'

Age group, years	KCMC crude cases	Q fever IFA performance multiplier*	KCMC hospital multiplier	KCMC adjusted cases	MRRH inpatient crude cases	MRRH outpatient crude cases	Sum of MRRH cases	Q fever IFA performance multiplier*	MRRH hospital multiplier	MRRH adjusted cases	Total adjusted cases‡	Paired sera	Enrollment logistics multiplier§	Annual Cases	Population	Annual Incidence per 100,000 persons
0-4	N/A	0.95	11.65	N/A	7	13	20	0.95	2.96	56	56	2.43	1.29	177	70807	250
5-14	N/A	0.95	36.10	N/A	6	1	7	0.95	2.64	18	18	1.28	1.29	29	155528	19
≥15	5	0.95	23.14	110	17	3	20	0.95	2.71	52	81	1.52	1.29	159	424694	37
Overall														365	651029	56
Age group, years	KCMC crude cases	SFGR IFA performance multiplier†	KCMC hospital multiplier	KCMC adjusted cases	MRRH inpatient crude cases	MRRH outpatient crude cases	Sum of MRRH cases	SFGR IFA performance multiplier†	MRRH hospital multiplier	MRRH adjusted cases	Total adjusted cases‡	Paired sera	Enrollment logistics multiplier§	Annual Cases	Population	Annual Incidence per 100,000 persons
0-4	N/A	1.06	11.65	N/A	13	15	28	1.06	2.96	88	88	2.43	1.29	276	70807	390
5-14	N/A	1.06	36.10	N/A	1	1	2	1.06	2.64	6	6	1.28	1.29	9	155528	6
≥15	6	1.06	23.14	148	14	7	21	1.06	2.71	61	104	1.52	1.29	205	424694	48
Overall														490	651029	75

* Q fever IFA performance multiplier was the specificity (95%) multiplied by the inverse of the sensitivity (100%).

† SFGR IFA performance multiplier was the specificity (100%) multiplied by the inverse of the sensitivity (94%).

‡ Total adjusted cases for those ≥15 years old was the sum of KCMC and MRRH cases divided by 2 to correct for recruitment of participants from two sentinel sites. Participants who were 0-4 years old and 5-14 years old were only recruited from one sentinel site, MRRH; thus, the total adjusted cases for these two age categories was the same as MRRH adjusted cases for these two age categories.

§ Enrollment logistics multiplier was the product of three distinct multipliers related to enrollment procedures: time multiplier, enrollment multiplier, and study duration multiplier. For 2012-2014, time multiplier was 1.4, enrollment multiplier was 2.09, and study duration multiplier was 0.44.

Abbreviations: IFA, indirect immunofluorescence assay; SFGR, spotted fever group rickettsioses; KCMC, Kilimanjaro Christian Medical Centre; MRRH, Mawenzi Regional Referral Hospital
